# Expression of Lipid Metabolism-Related Proteins in Metastatic Breast Cancer

**DOI:** 10.1371/journal.pone.0137204

**Published:** 2015-09-03

**Authors:** Yoon Yang Jung, Hye Min Kim, Ja Seung Koo

**Affiliations:** Department of Pathology, Yonsei University College of Medicine, Seoul, South Korea; Florida International University, UNITED STATES

## Abstract

**Purpose:**

The tumor biology of metastatic breast cancers differ according to the metastatic sites, and the features of cancer metabolism may also be different. The aim of this study is to investigate the expression of lipid metabolism-related proteins in metastatic breast cancer according to metastatic site and discuss the clinical significance thereof.

**Methods:**

Immunohistochemical staining for lipid metabolism-related proteins [fatty acid synthase (FASN), hormone-sensitive lipase (HSL), carnitine palmitoyltransferase IA (CPT-1A), acyl-CoA oxidase 1 (ACOX1), fatty acid binding protein 4 (FABP4,) and perilipin 1 (PLIN1)] was performed using a tissue microarray of 149 cases of metastatic breast cancer (bone metastasis = 39, brain metastasis = 37, liver metastasis = 21, and lung metastasis = 52).

**Results:**

The expression levels of ACOX1 (*p* = 0.009) and FASN (*p* = 0.007) varied significantly according to metastatic site, with the highest expression in brain metastasis and the lowest expression in liver metastasis. ACOX1 positivity (*p* = 0.005) and FASN positivity (*p* = 0.003) correlated with HER-2 positivity. The expression of FASN was significantly higher in HER-2 type breast cancer, and lower in luminal A and TNBC type breast cancer (*p*<0.001). Among lipid metabolism-related proteins, PLIN1 positivity was found to be an independent poor prognostic factor on multivariate analysis (Hazard ratio: 4.979, 95% CI: 1.054–22.59, *p* = 0.043).

**Conclusion:**

Different expression levels of lipid metabolism-related proteins were observed according to metastatic site. The expression of ACOX1 and FASN was highest in brain metastasis. These results suggest that the metastatic site should be considered when using lipid metabolism inhibitors for targeted therapy.

## Introduction

Breast cancer has a high mortality and morbidity, and distant metastasis is often responsible for the high mortality and morbidity. The major distant metastatic sites of breast cancer are lung, bone, brain, and liver [1,2], and among those, bone and brain metastases [3–8] are the most investigated. Multiple factors affect cancer progression, but the reciprocal interactions between tumor cells and host tissue are essential. Therefore, the interaction of cancer cells and distant organ tissue is expected to be important in distant metastasis.

The “seed and soil” hypothesis has been proposed for cancer metastasis, as specific carcinomas exhibit characteristic metastatic patterns. Breast cancer also exhibits unique characteristics according to its metastatic sites. Brain metastasis is associated with estrogen receptor (ER) negativity, HER-2/EGRF overexpression, and basal subtype [5–7], whereas bone metastasis is associated with ER positivity, and ER positivity/progesterone receptor (PR) negativity [4,9,10].

In tumors, a metabolic shift in energy production occurs, from oxidative phosphorylation in normal cells towards aerobic glycosis in cancer cells, which is called the Warburg effect [11]. However, different metabolic mechanisms may be used for energy production depending upon the tumor type [12]. One of these metabolic pathways is lipid metabolism; it includes lipid synthesis, lipid degradation and catabolism, and fatty acid (FA) oxidation. Fatty acid synthase (FASN) is one enzyme that is involved in lipid synthesis [13], whereas hormone-sensitive lipase (HSL) [13–15] is involved in lipid degradation and catabolism. Carnitine palmitoyltransferase IA (CPT-1A) and acyl-CoA oxidase 1 (ACOX1) are major enzymes involved in FA oxidation [16–18]. In addition, lipid transport and uptake is also an important process in lipid metabolism in cancer. Proteins involved in this process include fatty acid binding protein 4 (FABP4) and perilipin 1 (PLIN1) [19]. Because metastatic breast cancer exhibits unique characteristics depending upon its metastatic organs, it is reasonable to assume that they have different metabolic features, but this subject has rarely been studied thus far. This study aims to investigate variation in the expression of lipid metabolism-related proteins in different metastatic sites, and to discuss its clinical significance.

## Materials and Methods

### Patient selection

This study was approved by the Institutional Review Board (IRB) of Severance Hospital. The informed consent form was waived by IRB. Patient records/information was anonymized and de-identified prior to analysis. Patients with invasive primary breast cancer and metastasis to distant organs (lung, bone, brain, and liver) were selected from medical records of the Department of Pathology of Severance Hospital. Only patients with a diagnosis of invasive ductal carcinoma were included. In total, 149 cases were identified, and 36 cases were paired between primary cancer and metastatic cancer. All slides were reviewed, and pathologic diagnoses were approved by two pathologists (JSK and WHJ). Histological grade was assessed using the Nottingham grading system [20].

### Tissue microarray

Representative areas were selected on the H&E-stained slides of the tumors, and the corresponding spots were marked on the surfaces of the corresponding paraffin blocks. Using a biopsy needle, a 3-mm tissue core in the selected area was punched out and placed onto a 6 × 5 recipient block. Two tissue cores were extracted to minimize extraction bias. Each tissue core was assigned a unique tissue microarray location number that was linked to a database containing other clinicopathologic data.

### Immunohistochemistry (IHC)

The antibodies used for IHC in this study are shown in Table 1. Formalin-fixed, paraffin-embedded (FFPE) tissue samples were used as follows. Three-micron-thick slices from the FFPE tissue blocks were deparaffinized and rehydrated in xylene and alcohol solutions and stained using a Ventana Discovery XT automated stainer (Ventana Medical Systems, Tucson, AZ, USA). Antigen retrieval was performed with CC1 (Cell Conditioning 1) buffer (citrate buffer pH 6.0, Ventana Medical Systems). Appropriate positive and negative controls were used.

**Table 1:** Source, clone, and dilution of antibodies used in this study.

Antibody	Clone	Catalog number	Dilution	Company
*Molecular subtype-related*				
ER	SP1	RM-9101-S	1:100	Thermo Scientific, CA, USA
PR	PgR 636	M3569	1:50	DAKO, Denmark
HER-2	Polyclonal	A0485	1:1,500	DAKO, Denmark
Ki-67	MIB-1	M7240	1:150	DAKO, Denmark
*Lipolysis-related*				
HSL	Polyclonal	ab45422	1:100	Abcam, Cambridge, UK
PLIN1	Polyclonal	ab61682	1:100	Abcam, Cambridge, UK
FABP4	Polyclonal	ab13979	1:100	Abcam, Cambridge, UK
CPT-1	8F6AE9	ab128568	1:200	Abcam, Cambridge, UK
Acyl-CoA oxidase 1	Polyclonal	ab128549	1:50	Abcam, Cambridge, UK
FASN	EPR7466	ab128870	1:200	Abcam, Cambridge, UK

### Interpretation of immunohistochemical results

A cut-off value of 1% or higher positively-staining nuclei was used to define ER- and AR-positivity [21]. HER-2 staining was analyzed according to the American Society of Clinical Oncology (ASCO)/College of American Pathologists (CAP) guidelines using the following categories: 0 = no immunostaining; 1+ = weak incomplete membranous staining, less than 10% of tumor cells; 2+ = complete membranous staining, either uniform or weak in at least 10% of tumor cells; and 3+ = uniform intense membranous staining in at least 30% of tumor cells [22]. HER-2 staining was considered positive when strong (3+) membranous staining was observed, whereas it was considered negative when no or weak (0 to 1+) staining was noted.

The status of all immunohistochemical markers was determined using light microscopy to assess fractions of stained cells. HSL, PLIN1, FABP4, CPT-1A, ACOX1, and fatty acid synthase (FASN) immunostaining were scored as the product of the proportion of stained cells (0 = no staining, 1 = less than 30%, or 2 = more than 30%) and staining intensity (0 = no staining, 1 = weak, 2 = moderate, or 3 = strong). A total score of 2–6 was considered positive, while a score of 0 or 1 was considered negative [23].

### Tumor phenotype classification

In this study, breast cancer phenotypes were classified according to IHC results for ER, PR, HER-2, and Ki-67, as well as FISH results for HER-2 as follows [24]: *luminal A type*: ER and/or PR positive, HER-2 negative, and Ki-67 LI <14%; *luminal B type*: (HER-2 negative) ER and/or PR positive, HER-2 negative, and Ki-67 LI ≥14%, or (HER-2 positive) ER and/or PR positive and HER-2 overexpressing and/or amplified; *HER-2 type*: ER and PR negative and HER-2 overexpressing and/or amplified; and *triple negative breast cancer* (*TNBC) type*: ER, PR, and HER-2 negative.

### Statistical analysis

Data were statistically processed using SPSS for Windows, version 12.0 (SPSS Inc., Chicago, IL, USA). Correlation analysis of immunostaining results between primary breast cancer and metastatic breast cancer was calculated by the McNemar test. Student’s *t*- and Fisher’s exact tests were used to examine any differences in continuous and categorical variables, respectively. Corrected *p*-values and the Bonferroni method were used for multiple comparisons. Statistical significance was assumed when *P <*0.05. Kaplan-Meier survival curves and log-rank statistics were employed to evaluate time to tumor metastasis and time to survival. Multivariate regression analysis was performed using a Cox proportional hazards model.

## Results

### Baseline characteristics of patients

The baseline clinicopathologic characteristics of patients are summarized in Table 2. Among 149 patients with metastatic breast cancer, 39 (26.2%) had bone metastasis, 37 (24.8%) had brain metastasis, 21 (14.1%) had liver metastasis, and 52 (34.9%) had lung metastasis. The rate of ER positivity and PR positivity was higher in tumors of bone metastasis and liver metastasis (*p*<0.001), while brain metastatic tumors had a higher rate of HER-2 positivity (*p* = 0.017). The proportion of luminal A type tumors was significantly higher in bone metastasis and liver metastasis, while the rate of TNBC tumors was significantly higher in lung metastasis (*p*<0.001).

**Table 2:** Basal clinicopathologic characteristics of patients with breast cancer metastasis according to metastatic site.

Parameters		Total	Bone metastasis	Brain metastasis	Liver metastasis	Lung metastasis	*p*-value
		N = 149 (%)	n = 39 (%)	n = 37 (%)	n = 21(%)	n = 52 (%)	
Age (yr, mean±SD)		52.0±10.5	53.1±9.8	53.2±12.0	53.8±11.0	49.7±9.5	0.287*
ER							**<0.001** †
	Negative	68 (45.6)	7 (17.9)	26 (70.3)	6 (28.6)	29 (55.8)	
	Positive	81 (54.4)	32 (82.1)	11 (29.7)	15 (71.4)	23 (44.2)	
PR							**<0.001** †
	Negative	103 (69.1)	21 (53.8)	36 (97.3)	10 (47.6)	36 (69.2)	
	Positive	46 (30.9)	18 (46.2)	1 (2.7)	11 (52.4)	16 (30.8)	
HER-2							**0.017** †
	Negative	105 (70.5)	32 (82.1)	19 (51.4)	17 (81.0)	37 (71.2)	
	Positive	44 (29.5)	7 (17.9)	18 (48.6)	4 (19.0)	15 (28.8)	
Ki-67 LI							**0.001** †
	≤14	104 (69.8)	35 (89.7)	19 (51.4)	17 (81.0)	33 (63.5)	
	>14	45 (30.2)	4 (10.3)	18 (48.6)	4 (19.0)	19 (36.5)	
Molecular subtypes							**<0.001** †
	Luminal A	58 (38.9)	28 (71.8)	3 (8.1)	12 (57.1)	15 (28.8)	
	Luminal B	24 (16.1)	5 (12.8)	8 (21.6)	3 (14.3)	8 (15.4)	
	HER-2	29 (19.5)	4 (10.3)	12 (32.4)	3 (14.3)	10 (19.2)	
	TNBC	38 (25.5)	2 (5.1)	14 (37.8)	3 (14.3)	19 (36.5)	
Patient mortality		51 (34.2)	21 (53.8)	11 (29.7)	7 (33.3)	12 (23.1)	**0.020** †

* p-value was calculated by student’s t-test.

† p-value was calculated by Fisher’s exact test.

### Expression of lipid metabolism-related proteins in breast cancer metastasis according to metastatic site

**Fig 1:**
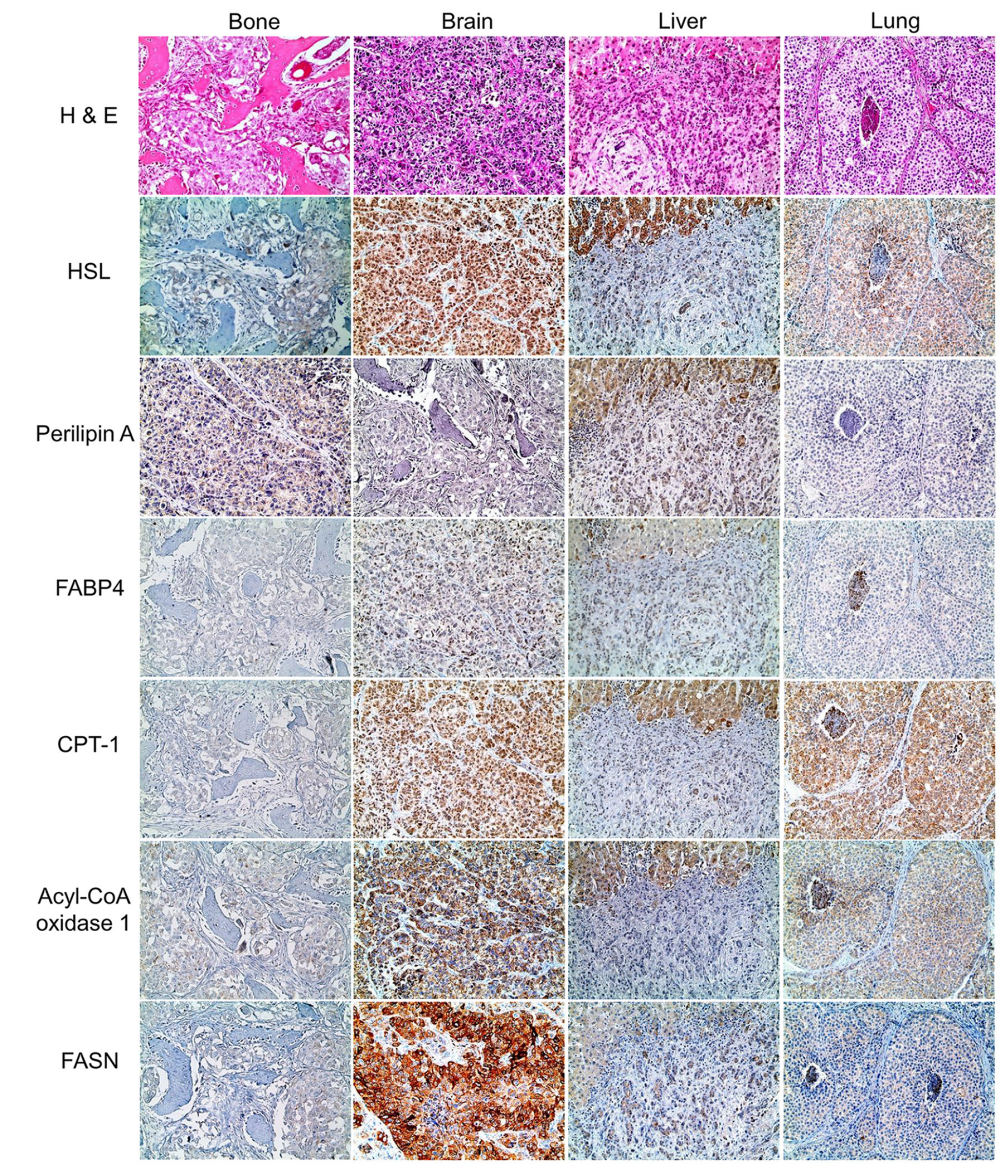
Expression of lipid metabolism-related proteins in metastatic breast cancer according to metastatic site. There were significant differences in the expression of lipid metabolism-related proteins according to metastatic site, with the highest expression of acyl-CoA oxidase 1 and FASN in brain metastases.

The expression of metabolism-related proteins according to metastatic site is summarized in Table 3 and Table A in S1 File. In an analysis of lipid metabolism-related protein expression according to metastatic site, the expression of ACOX1 (*p* = 0.009) and FASN (*p* = 0.007) varied significantly according to metastatic site, with the highest expression rates in brain metastasis, and the lowest expression rates in liver metastasis (Fig 1).

**Table 3:** Expression of metabolism-related proteins in the tumor cell compartment of breast cancer metastases according to metastatic site.

Parameters		Total	Bone metastasis	Brain metastasis	Liver metastasis	Lung metastasis	*p*-value*
		N = 149 (%)	n = 39 (%)	n = 37 (%)	n = 21(%)	n = 52 (%)	
HSL							0.199
	Negative	102 (68.5)	23 (59.0)	25 (67.6)	13 (61.9)	41 (78.8)	
	Positive	47 (31.5)	16 (41.0)	12 (32.4)	8 (38.1)	11 (21.2)	
PLIN1							0.388
	Negative	147 (98.7)	39 (100.0)	37 (100.0)	20 (95.2)	51 (98.1)	
	Positive	2 (1.3)	0 (0.0)	0 (0.0)	1 (4.8)	1 (1.9)	
FABP4							0.434
	Negative	142 (95.3)	36 (92.3)	37 (100.0)	20 (95.2)	49 (94.2)	
	Positive	7 (4.7)	3 (7.7)	0 (0.0)	1 (4.8)	3 (5.8)	
CPT-1A							0.381
	Negative	124 (83.2)	30 (76.9)	33 (89.2)	19 (90.5)	42 (80.8)	
	Positive	25 (16.8)	9 (23.1)	4 (10.8)	2 (9.5)	10 (19.2)	
Acyl-CoA oxidase 1							**0.009**
	Negative	111 (74.5)	30 (76.9)	20 (54.1)	18 (85.7)	43 (82.7)	
	Positive	38 (25.5)	9 (23.1)	17 (45.9)	3 (14.3)	9 (17.3)	
FASN							**0.007**
	Negative	105 (70.5)	30 (76.9)	18 (48.6)	18 (85.7)	39 (75.0)	
	Positive	44 (29.5)	9 (23.1)	19 (51.4)	3 (14.3)	13 (25.0)	

* p-value was calculated by Fisher’s exact test.

### Correlation of expression of lipid metabolism-related proteins between primary and metastatic breast cancer according to metastatic site

We investigated lipid metabolism-related protein expression in 36 cases of paired primary and metastatic cancer. The results are shown in Table 4 and Table B in S1 File. FASN expression was significantly different between primary and metastatic cancer (*p* = 0.008). In 8 (22.2%) of the paired cases, the primary tumor was FASN-positive, while the metastatic tumor was FASN-negative.

**Table 4:** Correlation of expression of metabolism-related proteins between primary and metastatic breast cancer according to metastatic site^*^.

Parameters		Total		Bone metastasis		Brain metastasis		Liver metastasis		Lung metastasis	
		N = 36 (%)	*p*-value	n = 8 (%)	*p*-value	n = 5 (%)	*p*-value	n = 2 (%)	*p*-value	n = 21 (%)	*p*-value
HSL			0.125		1.000		1.000		1.000		0.125
	(+) → (+)	6 (16.7)		4 (50.0)		0 (0.0)		1 (50.0)		1 (4.8)	
	(+) → (-)	6 (16.7)		1 (12.5)		1 (20.0)		0 (0.0)		4 (19.0)	
	(-) → (+)	1 (2.8)		0 (0.0)		1 (20.0)		0 (0.0)		0 (0.0)	
	(-) → (-)	23 (63.9)		3 (37.5)		3 (60.0)		1 (50.0)		16 (76.2)	
CPT-1A			0.500		1.000		1.000		N/A		**N/A**
	(+) → (+)	2 (5.6)		0 (0.0)		2 (40.0)		0 (0.0)		0 (0.0)	
	(+) → (-)	2 (5.6)		1 (12.5)		1 (20.0)		0 (0.0)		0 (0.0)	
	(-) → (+)	0 (0.0)		0 (0.0)		0 (0.0)		0 (0.0)		0 (0.0)	
	(-) → (-)	32 (88.9)		7 (87.5)		2 (40.0)		2 (100.0)		21 (100.0)	
Acyl-CoA oxidase 1			0.125		N/A		1.000		1.000		0.375
	(+) → (+)	2 (5.6)		0 (0.0)		1 (20.0)		0 (0.0)		1 (4.8)	
	(+) → (-)	6 (16.7)		0 (0.0)		1 (20.0)		1 (50.0)		4 (19.0)	
	(-) → (+)	1 (2.8)		0 (0.0)		0 (0.0)		0 (0.0)		1 (4.8)	
	(-) → (-)	27 (75.0)		8 (100.0)		3 (60.0)		1 (50.0)		15 (71.4)	
FASN			**0.008**		0.500		1.000		1.000		0.125
	(+) → (+)	2 (5.6)		0 (0.0)		2 (40.0)		0 (0.0)		0 (0.0)	
	(+) → (-)	8 (22.2)		2 (25.0)		1 (20.0)		1 (50.0)		4 (19.0)	
	(-) → (+)	0 (0.0)		0 (0.0)		0 (0.0)		0 (0.0)		0 (0.0)	
	(-) → (-)	26 (72.2)		6 (75.0)		2 (40.0)		1 (50.0)		17 (81.0)	

* The data of FABP4 and PLIN1were removed because almost all cases were negative for FABP4 and PLIN1.

### Correlation between pathologic factors and expression of lipid metabolism-related proteins

**Fig 2:**
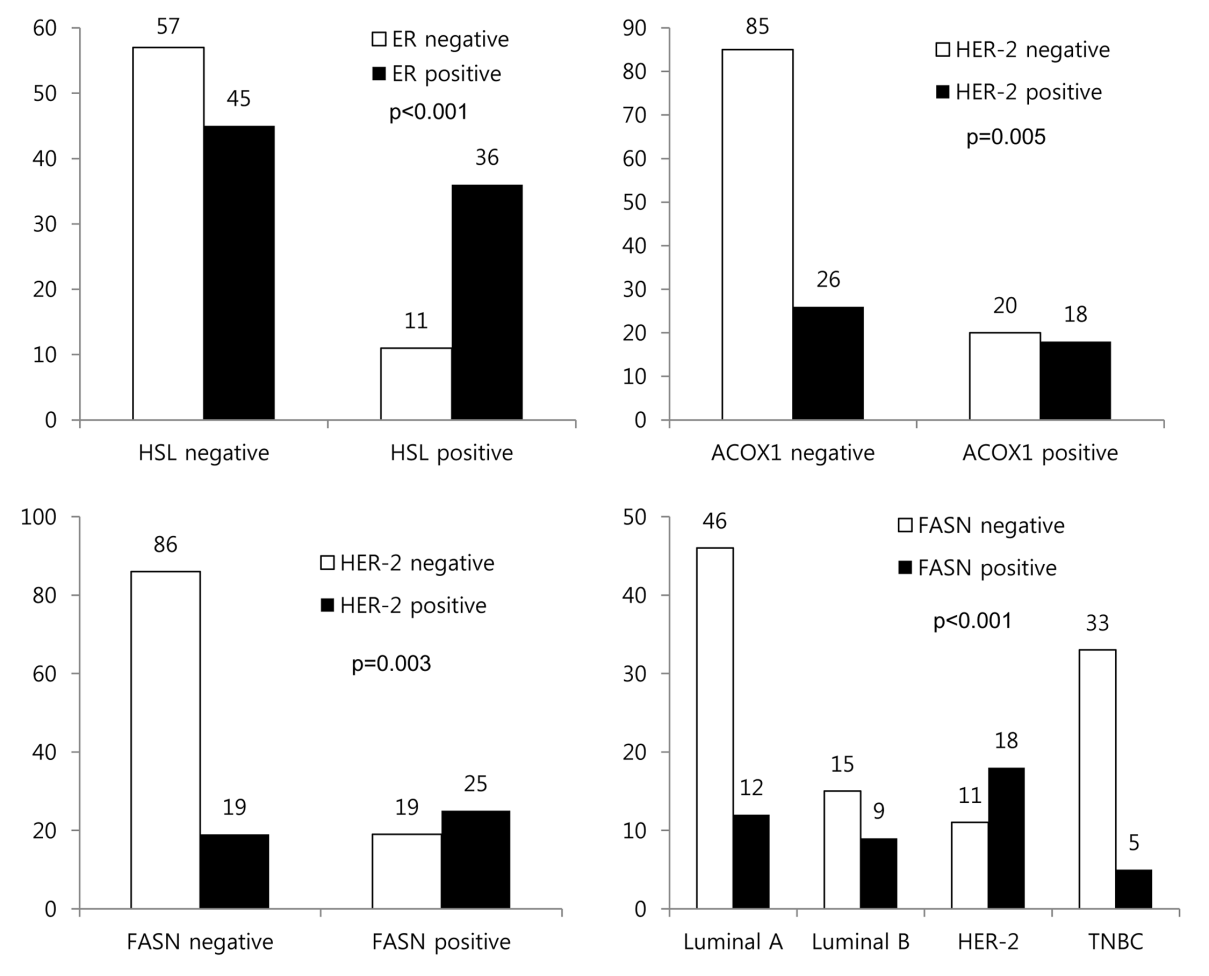
Correlation between pathologic factors and expression of lipid metabolism-related proteins.

In an analysis of pathologic factors and lipid metabolism-related protein expression, ER positivity correlated with HSL positivity (*p*<0.001). HER-2 positivity was associated with ACOX1 positivity (*p* = 0.005) and FASN positivity (*p* = 0.003). FASN expression varied according to molecular subtype (*p*<0.001), with higher expression in HER-2 types and lower expression in luminal A types and TNBCs (Fig 2).

### Association between the expression of lipid metabolism-related proteins and patient prognosis

**Fig 3:**
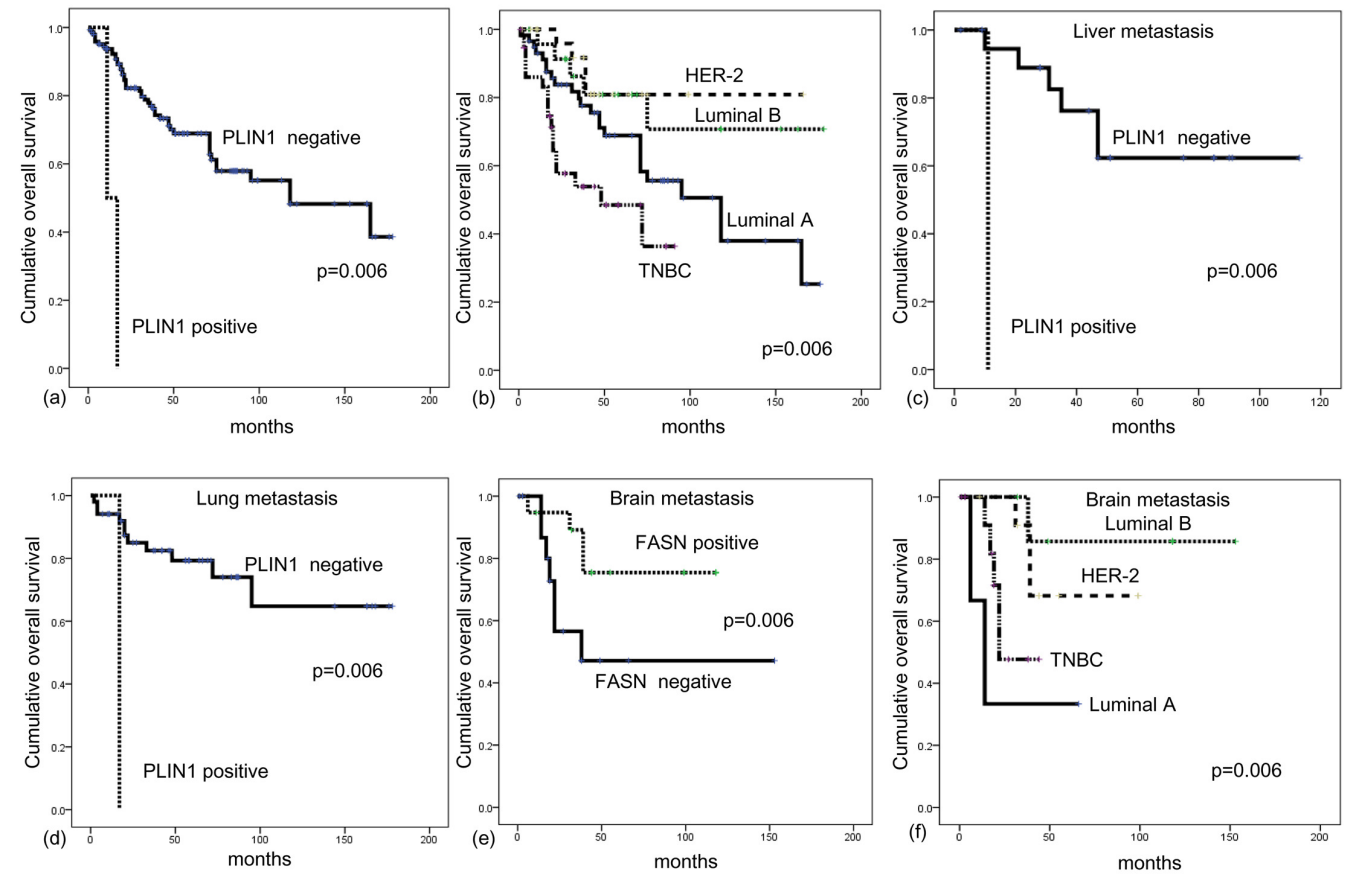
The impact of expression of lipid metabolism related proteins in metastatic breast cancer (a, b), liver metastasis (c), lung metastasis (d) and brain metastasis (e, f).

A univariate analysis was performed to analyze the association between the expression of lipid metabolism-related proteins and patient prognosis (Table 5). Factors associated with shorter overall survival (OS) included PLIN1 positivity (*p*<0.001) and molecular subtype (TNBC, *p* = 0.002). In an analysis according to metastatic site, FASN negativity (*p* = 0.039) was significantly associated with shorter OS in brain metastases, and PLIN1 positivity (*p* = 0.005) correlated with liver and lung metastasis (Fig 3).

**Table 5:** Univariate analysis of the association between expression levels of metabolism-related proteins in metastatic breast cancers and overall survival by the log-rank test.

Parameters		Total		Bone metastasis		Brain metastasis		Liver metastasis		Lung metastasis	
		N = 149 (%)		n = 39 (%)		n = 37 (%)		n = 21 (%)		n = 52 (%)	
		Mean survival (95% CI) months	*P*-value	Mean survival (95% CI) months	*P*-value	Mean survival (95% CI) months	*P*-value	Mean survival (95% CI) months	*P*-value	Mean survival (95% CI) months	*P*-value
HSL			0.497		0.762		0.259		0.634		0.672
	Negative	105 (88–123)		81 (53–110)		95 (66–123)		83 (59–106)		125 (98–153)	
	Positive	112 (90–134)		78 (52–104)		98 (73–122)		62 (37–86)		140 (106–174)	
PLIN1			**<0.001**		N/A		N/A		**0.005**		**0.005**
	Negative	111 (96–125)		N/A		N/A		82 (63–102)		132 (108–156)	
	Positive	14 (8–19)		N/A		N/A		11 (11–11)		17 (17–17)	
FABP4			0.920		0.439		N/A		N/A		0.746
	Negative	109 (94–123)		83 (62–105)		N/A		N/A		130 (105–154)	
	Positive	62 (36–89)		19 (14–24)		N/A		N/A		63 (26–99)	
CPT-1A			0.880		0.714		N//A		0.208		0.660
	Negative	111 (96–126)		85 (61–110)		N/A		82 (61–102)		134 (109–159)	
	Positive	66 (55–78)		59 (38–80)		N/A		35 (35–35)		69 (51–87)	
Acyl-CoA oxidase 1			0.968		0.740		0.165		0.131		0.870
	Negative	113 (96–130)		91 (62–120)		94 (61–127)		84 (64–104)		129 (103–156)	
	Positive	100 (79–122)		78 (55–101)		92 (70–113)		26 (9–43)		124 (80–169)	
FASN			0.127		0.553		**0.039**		0.914		0.473
	Negative	101 (84–118)		85 (62–109)		83 (47–119)		78 (57–99)		124 (96–151)	
	Positive	91 (78–105)		53 (30–76)		96 (77–115)		60 (20–99)		76 (62–89)	
Molecular subtypes			**0.002**		**N/A**		**0.034**		**N/A**		**N/A**
	Luminal A	102 (83–122)		N/A		28 (0–58)		N/A		N/A	
	Luminal B	138 (108–168)		N/A		136 (106–166)		N/A		N/A	
	HER-2	140 (118–163)		N/A		79 (60–97)		N/A		N/A	
	TNBC	51 (38–64)		N/A		31 (22–39)		N/A		N/A	

In a multivariate analysis, higher KI-67 LI (Hazard ratio: 2.913, 95% CI: 1.313–6.462, *p* = 0.009), and PLIN1 positivity (Hazard ratio: 4.979, 95% CI: 1.054–22.59, *p* = 0.043) showed significant associations with shorter OS. The results are shown in Table 6.

**Table 6:** Multivariate analysis of patient prognoses in metastatic breast cancer.

Parameters	Overall survival		
	Hazard ratio	95% CI	*P*-value
ER **negative** vs. positive	0.992	0.226–4.360	0.992
PR **negative** vs. Positive	1.377	0.651–2.910	0.403
HER-2 negative vs. **positive**	0.493	0.146–1.667	0.255
Ki-67 LI ≤14 vs. **>14**	2.913	1.313–6.462	**0.009**
PLIN1 negative vs. **positive**	4.879	1.054–22.59	**0.043**
Non-TNBC vs. **TNBC**	2.815	0.548–14.47	0.215

Bold: shorter overall survival

## Discussion

In this study, we observed a significant difference in lipid metabolism-related protein expression in metastatic breast cancers according to metastatic site. The expression of ACOX1 (*p* = 0.009) and FASN (*p* = 0.007) was higher in brain metastasis and lower in liver metastasis. One possible explanation for this lies in differences in cancer cell characteristics according to metastatic site. In our study, molecular subtype differed according to metastatic site, with a higher rate of HER-2 type in brain metastasis, and a higher rate of luminal A type in liver metastasis. In a previous study, FASN expression was reported to be associated with HER-2 expression [25]. Our study found an association between HER-2 positivity and FASN positivity, which is in agreement with the previous study. In a study on the molecular interaction between HER-2 and FASN, it was reported that phosphorylation of FASN occurs when it is in a complex with HER-2, which results in an increase in FASN enzymatic activity [26], suggesting an axis between HER-2 and FASN that accelerates cancer cell proliferation, survival, and metastasis. Another possible explanation could lie in the differing environments of the metastatic sites. The brain, unlike other organs, produces energy by glycolysis using glucose, not by mitochondrial oxidative phosphorylation, and thus the metabolic environment of the brain is different from that of other organs. It has been reported that metabolism in the brain, such as fatty acid oxidation, is different from that in other tissues [27]. Thus, this difference in metabolic environments could be an explanation for the differences in metabolic features, but further study would be required to evaluate this hypothesis.

Our results showed that FASN expression was significantly different between primary and metastatic tumors, and in most cases, the shift was from FASN-positivity in primary tumors to FASN-negativity in metastatic tumors. Discordance between primary and metastatic tumors has been also reported for ER, PR, and HER-2, which are the most important biomarkers of breast cancer. HER-2 loss in 21–50% and HER-2 gain in 30% of metastatic tumors [8,28], ER loss in 3.2–44% of metastatic tumors [29–31], and PR loss in 24% of metastatic tumors [31] have been reported, whereas no gain of ER or PR in metastatic tumors have been reported. The discordance in FASN expression between primary and metastatic tumors found in our study could be explained in a similar context as the aforementioned studies, but further study is required to explore its clinical meaning.

In the present study, PLIN1 positivity of metastatic breast cancer was found to be an independent prognostic factor. In previous studies, an association of PLIN1 and prognosis has rarely been reported. PLIN1 is a lipid droplet associated protein, and is reported to act as a lipid droplet gate-keeper [19]. In breast cancer, lipid droplet formation is reported to be associated with prolonged breast cancer survival [32], so this could explain our result of PLIN1 positivity and its association with poor prognosis. But in this study, only a small proportion (1.3%) of metastatic breast cancer had positive results and there may be limitations for a statistical analysis. A further study involving a large volume of metastatic breast cancer may be needed.

The clinical significance of this study is the potential for lipid metabolism pathway inhibition to serve as a pharmaceutical treatment target. With respect to lipid metabolism, inhibition of FASN has been reported to inhibit tumor growth [26,33–36]. In addition, small molecules that inhibit fatty acid synthesis-related enzymes, such as acetyl-CoA carboxylase, are suggested candidate for cancer therapy [37], a further study is needed to validate the possibility of lipid metabolism molecules as treatment targets.

In our previous study, the expression of lipid metabolism-related proteins that were evaluated in the present study had been investigated in primary breast cancers. The expression of lipid metabolism-related proteins varied according to the molecular subtype defined by surrogate immunohistochemistry [38]. And the present study showed differences of lipid-metabolism related proteins according to metastatic site. These results suggest the different lipid metabolism of breast cancers in various conditions.

In conclusion, our study revealed differences in lipid metabolism-related protein expression according to metastatic site, and found that the degrees of ACOX1 and FASN expression were highest in brain metastasis, and lowest in liver metastasis.

## Supporting Information

S1 File Raw data of expression of lipid metabolism related proteins in metastatic breast cancer (Table A). Raw data of expression of lipid metabolism related proteins in paired primary and metastatic breast cancer (Table B).
(DOCX)
