# Supplementary material for: Expression of Lipid Metabolism-Related Proteins in Metastatic Breast Cancer
Source: PLoS One. 2015 Sep 3;10(9):e0137204. doi: 10.1371/journal.pone.0137204 (PMC4559312; doi:10.1371/journal.pone.0137204)
Supplement: S1 File — Raw data of expression of lipid metabolism related proteins in paired primary and metastatic breast cancer (Table B). (DOCX) [file pone.0137204.s001.docx]

Supporting Information files

| Table 1 Raw data of expression of lipid metabolism related proteins in metastatic breast cancer | | | | | | | | | | | | | | | | |
| --- | --- | --- | --- | --- | --- | --- | --- | --- | --- | --- | --- | --- | --- | --- | --- | --- |
| met site | Age | newER | newPR | newHER2 | Ki67 | Molecular  TYPE | OS | OStime | newAGE | newKI67 | newHSL | newPLIN | newFABP4 | newCPT1A | newACOX1 | newFASN |
| bone | 78 | 1 | 0 | 0 | 15 | B | 0 | 6 | 2 | 2 | 0 | 0 | 0 | 1 | 0 | 0 |
| bone | 78 | 1 | 0 | 0 | 1 | A | 0 | 6 | 2 | 1 | 1 | 0 | 1 | 1 | 0 | 0 |
| bone | 61 | 1 | 1 | 0 | 3 | A | 0 | 23 | 2 | 1 | 1 | 0 | 1 | 1 | 0 | 0 |
| bone | 49 | 1 | 0 | 0 | 1 | A | 1 | 71 | 1 | 1 | 0 | 0 | 0 | 0 | 1 | 0 |
| bone | 49 | 1 | 0 | 0 | 1 | A | 1 | 71 | 1 | 1 | 0 | 0 | 0 | 0 | 1 | 0 |
| bone | 49 | 1 | 0 | 0 | 1 | A | 1 | 71 | 1 | 1 | 0 | 0 | 0 | 0 | 1 | 0 |
| bone | 49 | 1 | 0 | 0 | 5 | A | 1 | 71 | 1 | 1 | 0 | 0 | 0 | 0 | 1 | 0 |
| bone | 57 | 1 | 0 | 0 | 1 | A | 0 | 51 | 2 | 1 | 1 | 0 | 0 | 1 | 1 | 1 |
| bone | 57 | 1 | 0 | 0 | 1 | A | 0 | 51 | 2 | 1 | 1 | 0 | 0 | 1 | 1 | 1 |
| bone | 46 | 1 | 1 | 0 | 1 | A | 0 | 84 | 1 | 1 | 1 | 0 | 0 | 0 | 0 | 0 |
| bone | 51 | 1 | 1 | 0 | 1 | A | 0 | 37 | 2 | 1 | 0 | 0 | 0 | 0 | 0 | 0 |
| bone | 60 | 1 | 1 | 0 | 1 | A | 1 | 21 | 2 | 1 | 0 | 0 | 0 | 0 | 0 | 0 |
| bone | 52 | 1 | 0 | 0 | 0 | A | 0 | 122 | 2 | 1 | 0 | 0 | 0 | 0 | 0 | 0 |
| bone | 47 | 1 | 1 | 0 | 0 | A | 1 | 165 | 1 | 1 | 0 | 0 | 0 | 0 | 0 | 0 |
| bone | 45 | 1 | 0 | 0 | 0 | A | 1 | 16 | 1 | 1 | 1 | 0 | 1 | 0 | 1 | 1 |
| bone | 57 | 1 | 0 | 0 | 1 | A | 1 | 36 | 2 | 1 | 1 | 0 | 0 | 0 | 0 | 0 |
| bone | 44 | 1 | 0 | 0 | 20 | B | 0 | 27 | 1 | 2 | 0 | 0 | 0 | 0 | 0 | 0 |
| bone | 44 | 1 | 0 | 0 | 20 | B | 0 | 27 | 1 | 2 | 0 | 0 | 0 | 0 | 0 | 0 |
| bone | 48 | 1 | 0 | 0 | 0 | A | 0 | 96 | 1 | 1 | 1 | 0 | 0 | 0 | 0 | 0 |
| bone | 75 | 1 | 1 | 0 | 0 | A | 0 | 78 | 2 | 1 | 0 | 0 | 0 | 0 | 0 | 0 |
| bone | 65 | 0 | 0 | 1 | 50 | H | 0 | 38 | 2 | 2 | 0 | 0 | 0 | 0 | 0 | 1 |
| bone | 65 | 0 | 0 | 1 | 10 | H | 0 | 38 | 2 | 1 | 0 | 0 | 0 | 0 | 0 | 1 |
| bone | 50 | 1 | 1 | 0 | 10 | A | 1 | 75 | 1 | 1 | 1 | 0 | 0 | 1 | 0 | 1 |
| bone | 45 | 1 | 0 | 0 | 0 | A | 1 | 16 | 1 | 1 | 1 | 0 | 0 | 0 | 0 | 1 |
| bone | 49 | 1 | 1 | 1 | 5 | B | 1 | 75 | 1 | 1 | 0 | 0 | 0 | 1 | 0 | 0 |
| bone | 48 | 1 | 1 | 0 | 1 | A | 0 | 93 | 1 | 1 | 0 | 0 | 0 | 0 | 0 | 0 |
| bone | 49 | 1 | 1 | 0 | 0 | A | 1 | 118 | 1 | 1 | 1 | 0 | 0 | 0 | 1 | 0 |
| bone | 49 | 1 | 1 | 0 | 0 | A | 1 | 118 | 1 | 1 | 1 | 0 | 0 | 0 | 1 | 0 |
| bone | 50 | 1 | 1 | 0 | 0 | A | 1 | 9 | 1 | 1 | 0 | 0 | 0 | 0 | 0 | 0 |
| bone | 38 | 1 | 1 | 0 | 0 | A | 1 | 19 | 1 | 1 | 1 | 0 | 0 | 1 | 0 | 0 |
| bone | 58 | 1 | 1 | 0 | 0 | A | 1 | 1 | 2 | 1 | 1 | 0 | 0 | 0 | 0 | 1 |
| bone | 38 | 1 | 1 | 1 | 0 | A | 1 | 50 | 1 | 1 | 0 | 0 | 0 | 0 | 0 | 0 |
| bone | 44 | 0 | 0 | 0 | 0 | T | 1 | 4 | 1 | 1 | 0 | 0 | 0 | 0 | 0 | 0 |
| bone | 61 | 0 | 0 | 1 | 0 | H | 0 | 72 | 2 | 1 | 1 | 0 | 0 | 0 | 0 | 0 |
| bone | 40 | 0 | 1 | 1 | 0 | B | 1 | 30 | 1 | 1 | 0 | 0 | 0 | 1 | 0 | 0 |
| bone | 63 | 1 | 1 | 0 | 0 | A | 0 | 53 | 2 | 1 | 0 | 0 | 0 | 0 | 0 | 1 |
| bone | 55 | 1 | 1 | 0 | 0 | A | 1 | 42 | 2 | 1 | 0 | 0 | 0 | 0 | 0 | 0 |
| bone | 50 | 0 | 0 | 0 | 1 | T | 1 | 3 | 1 | 1 | 0 | 0 | 0 | 0 | 0 | 0 |
| bone | 61 | 0 | 0 | 1 | 0 | H | 0 | 72 | 2 | 1 | 1 | 0 | 0 | 0 | 0 | 0 |
| brain | 70 | 0 | 0 | 0 | 1 | T | 0 | 3 | 2 | 1 | 0 | 0 | 0 | 0 | 0 | 0 |
| brain | 56 | 0 | 0 | 0 | 10 | T | 1 | 19 | 2 | 1 | 0 | 0 | 0 | 0 | 0 | 0 |
| brain | 46 | 0 | 0 | 1 | 20 | H | 1 | 39 | 1 | 2 | 0 | 0 | 0 | 0 | 1 | 1 |
| brain | 46 | 0 | 0 | 1 | 12 | H | 1 | 39 | 1 | 1 | 0 | 0 | 0 | 0 | 1 | 1 |
| brain | 32 | 0 | 0 | 0 | 5 | T | 0 | 27 | 1 | 1 | 0 | 0 | 0 | 0 | 0 | 0 |
| brain | 46 | 0 | 0 | 0 | 1 | T | 0 | 19 | 1 | 1 | 0 | 0 | 0 | 0 | 1 | 0 |
| brain | 43 | 0 | 0 | 0 | 2 | T | 1 | 17 | 1 | 1 | 0 | 0 | 0 | 0 | 0 | 0 |
| brain | 70 | 1 | 0 | 0 | 2 | A | 1 | 14 | 2 | 1 | 0 | 0 | 0 | 0 | 0 | 0 |
| brain | 40 | 0 | 0 | 1 | 20 | H | 1 | 31 | 1 | 2 | 0 | 0 | 0 | 0 | 1 | 1 |
| brain | 29 | 0 | 0 | 0 | 5 | T | 0 | 44 | 1 | 1 | 0 | 0 | 0 | 1 | 0 | 1 |
| brain | 44 | 0 | 0 | 1 | 5 | H | 0 | 11 | 1 | 1 | 1 | 0 | 0 | 1 | 1 | 1 |
| brain | 49 | 1 | 0 | 0 | 0 | A | 1 | 6 | 1 | 1 | 1 | 0 | 0 | 0 | 0 | 1 |
| brain | 70 | 0 | 0 | 0 | 10 | T | 0 | 3 | 2 | 1 | 0 | 0 | 0 | 0 | 0 | 0 |
| brain | 55 | 0 | 0 | 0 | 30 | T | 0 | 38 | 2 | 2 | 1 | 0 | 0 | 0 | 0 | 0 |
| brain | 55 | 0 | 0 | 0 | 30 | T | 0 | 38 | 2 | 2 | 1 | 0 | 0 | 0 | 0 | 0 |
| brain | 58 | 0 | 0 | 1 | 25 | H | 0 | 55 | 2 | 2 | 0 | 0 | 0 | 1 | 0 | 1 |
| brain | 66 | 0 | 0 | 0 | 1 | T | 0 | 1 | 2 | 1 | 0 | 0 | 0 | 0 | 0 | 0 |
| brain | 70 | 1 | 0 | 1 | 40 | B | 0 | 118 | 2 | 2 | 1 | 0 | 0 | 0 | 1 | 1 |
| brain | 70 | 1 | 0 | 1 | 40 | B | 0 | 118 | 2 | 2 | 1 | 0 | 0 | 0 | 1 | 1 |
| brain | 70 | 1 | 0 | 1 | 40 | B | 0 | 118 | 2 | 2 | 1 | 0 | 0 | 0 | 1 | 1 |
| brain | 70 | 1 | 0 | 1 | 40 | B | 0 | 118 | 2 | 2 | 1 | 0 | 0 | 0 | 1 | 1 |
| brain | 47 | 1 | 0 | 0 | 10 | A | 0 | 66 | 1 | 1 | 0 | 0 | 0 | 0 | 0 | 0 |
| brain | 59 | 0 | 0 | 1 | 10 | H | 0 | 44 | 2 | 1 | 0 | 0 | 0 | 0 | 1 | 1 |
| brain | 59 | 0 | 0 | 1 | 10 | H | 0 | 44 | 2 | 1 | 0 | 0 | 0 | 0 | 1 | 1 |
| brain | 59 | 0 | 0 | 1 | 10 | H | 0 | 44 | 2 | 1 | 0 | 0 | 0 | 0 | 1 | 1 |
| brain | 33 | 0 | 0 | 0 | 25 | T | 0 | 18 | 1 | 2 | 0 | 0 | 0 | 0 | 0 | 0 |
| brain | 54 | 1 | 1 | 1 | 20 | B | 0 | 49 | 2 | 2 | 0 | 0 | 0 | 0 | 0 | 0 |
| brain | 43 | 0 | 0 | 0 | 2 | T | 1 | 14 | 1 | 1 | 0 | 0 | 0 | 0 | 0 | 0 |
| brain | 43 | 1 | 0 | 0 | 15 | B | 0 | 153 | 1 | 2 | 0 | 0 | 0 | 0 | 0 | 0 |
| brain | 53 | 0 | 0 | 1 | 20 | H | 0 | 32 | 2 | 2 | 1 | 0 | 0 | 0 | 1 | 1 |
| brain | 53 | 0 | 0 | 1 | 50 | H | 0 | 32 | 2 | 2 | 1 | 0 | 0 | 0 | 1 | 1 |
| brain | 69 | 0 | 0 | 1 | 2 | H | 0 | 99 | 2 | 1 | 0 | 0 | 0 | 0 | 1 | 1 |
| brain | 46 | 0 | 0 | 0 | 20 | T | 1 | 22 | 1 | 2 | 0 | 0 | 0 | 0 | 0 | 0 |
| brain | 46 | 0 | 0 | 0 | 20 | T | 1 | 22 | 1 | 2 | 0 | 0 | 0 | 0 | 0 | 0 |
| brain | 44 | 1 | 0 | 0 | 50 | B | 1 | 38 | 1 | 2 | 1 | 0 | 0 | 0 | 1 | 0 |
| brain | 42 | 1 | 0 | 1 | 30 | B | 0 | 32 | 1 | 2 | 1 | 0 | 0 | 1 | 1 | 1 |
| brain | 66 | 0 | 0 | 1 | 0 | H | 0 | 99 | 2 | 1 | 0 | 0 | 0 | 0 | 0 | 1 |
| liver | 49 | 1 | 1 | 0 | 1 | A | 1 | 10 | 1 | 1 | 1 | 0 | 0 | 0 | 0 | 0 |
| liver | 57 | 0 | 0 | 0 | 20 | T | 0 | 51 | 2 | 2 | 0 | 0 | 0 | 0 | 0 | 0 |
| liver | 57 | 0 | 0 | 0 | 20 | T | 0 | 51 | 2 | 2 | 0 | 0 | 0 | 0 | 0 | 0 |
| liver | 63 | 0 | 0 | 1 | 1 | H | 0 | 2 | 2 | 1 | 0 | 0 | 0 | 0 | 0 | 0 |
| liver | 55 | 1 | 0 | 0 | 1 | A | 0 | 9 | 2 | 1 | 1 | 0 | 0 | 1 | 0 | 0 |
| liver | 32 | 1 | 1 | 0 | 20 | B | 0 | 28 | 1 | 2 | 0 | 0 | 0 | 0 | 0 | 0 |
| liver | 32 | 1 | 1 | 0 | 1 | A | 0 | 28 | 1 | 1 | 0 | 0 | 0 | 0 | 0 | 0 |
| liver | 29 | 1 | 0 | 0 | 1 | A | 0 | 44 | 1 | 1 | 1 | 0 | 0 | 0 | 0 | 0 |
| liver | 56 | 1 | 1 | 0 | 1 | A | 1 | 35 | 2 | 1 | 1 | 0 | 0 | 1 | 0 | 0 |
| liver | 59 | 0 | 0 | 1 | 1 | H | 0 | 75 | 2 | 1 | 1 | 0 | 1 | 0 | 0 | 0 |
| liver | 54 | 1 | 1 | 0 | 1 | A | 0 | 85 | 2 | 1 | 0 | 0 | 0 | 0 | 0 | 1 |
| liver | 63 | 1 | 0 | 0 | 1 | A | 1 | 31 | 2 | 1 | 1 | 0 | 0 | 0 | 0 | 0 |
| liver | 67 | 0 | 0 | 1 | 1 | H | 0 | 47 | 2 | 1 | 0 | 0 | 0 | 0 | 1 | 0 |
| liver | 53 | 1 | 1 | 0 | 0 | A | 0 | 90 | 2 | 1 | 1 | 0 | 0 | 0 | 0 | 0 |
| liver | 66 | 1 | 0 | 0 | 50 | B | 1 | 11 | 2 | 2 | 0 | 1 | 0 | 0 | 1 | 1 |
| liver | 60 | 1 | 1 | 1 | 0 | B | 1 | 21 | 2 | 1 | 0 | 0 | 0 | 0 | 1 | 0 |
| liver | 54 | 1 | 1 | 0 | 1 | A | 0 | 85 | 2 | 1 | 1 | 0 | 0 | 0 | 0 | 1 |
| liver | 46 | 1 | 1 | 0 | 0 | A | 1 | 47 | 1 | 1 | 0 | 0 | 0 | 0 | 0 | 0 |
| liver | 46 | 1 | 1 | 0 | 0 | A | 1 | 47 | 1 | 1 | 0 | 0 | 0 | 0 | 0 | 0 |
| liver | 65 | 0 | 0 | 0 | 0 | T | 0 | 91 | 2 | 1 | 0 | 0 | 0 | 0 | 0 | 0 |
| liver | 58 | 1 | 1 | 0 | 0 | A | 0 | 113 | 2 | 1 | 0 | 0 | 0 | 0 | 0 | 0 |
| lung | 62 | 0 | 0 | 0 | 20 | T | 1 | 17 | 2 | 2 | 1 | 0 | 0 | 0 | 0 | 1 |
| lung | 54 | 0 | 0 | 1 | 7 | H | 0 | 38 | 2 | 1 | 0 | 0 | 0 | 1 | 1 | 1 |
| lung | 58 | 0 | 0 | 0 | 30 | T | 0 | 58 | 2 | 2 | 0 | 0 | 0 | 0 | 0 | 0 |
| lung | 58 | 1 | 0 | 0 | 30 | B | 0 | 58 | 2 | 2 | 1 | 0 | 0 | 0 | 1 | 0 |
| lung | 38 | 1 | 1 | 0 | 50 | B | 0 | 56 | 1 | 2 | 0 | 0 | 0 | 0 | 1 | 1 |
| lung | 45 | 1 | 1 | 0 | 1 | A | 0 | 25 | 1 | 1 | 0 | 0 | 0 | 0 | 0 | 0 |
| lung | 48 | 1 | 1 | 0 | 1 | A | 0 | 168 | 1 | 1 | 1 | 0 | 0 | 0 | 0 | 0 |
| lung | 51 | 0 | 0 | 0 | 70 | T | 0 | 58 | 2 | 2 | 0 | 0 | 0 | 0 | 0 | 0 |
| lung | 42 | 0 | 0 | 0 | 30 | T | 1 | 20 | 1 | 2 | 0 | 0 | 0 | 1 | 0 | 0 |
| lung | 42 | 0 | 0 | 0 | 1 | T | 1 | 20 | 1 | 1 | 0 | 0 | 0 | 1 | 0 | 0 |
| lung | 35 | 1 | 0 | 0 | 10 | A | 0 | 83 | 1 | 1 | 0 | 0 | 0 | 0 | 0 | 0 |
| lung | 52 | 1 | 1 | 0 | 1 | A | 0 | 20 | 2 | 1 | 1 | 0 | 0 | 0 | 0 | 0 |
| lung | 38 | 1 | 1 | 0 | 10 | A | 0 | 56 | 1 | 1 | 0 | 0 | 0 | 0 | 0 | 0 |
| lung | 64 | 0 | 0 | 1 | 3 | H | 0 | 11 | 2 | 1 | 0 | 0 | 0 | 0 | 0 | 0 |
| lung | 66 | 0 | 0 | 0 | 1 | T | 0 | 18 | 2 | 1 | 1 | 0 | 0 | 0 | 0 | 0 |
| lung | 48 | 1 | 0 | 0 | 1 | A | 0 | 11 | 1 | 1 | 0 | 0 | 0 | 0 | 0 | 0 |
| lung | 66 | 0 | 0 | 0 | 60 | T | 0 | 18 | 2 | 2 | 0 | 0 | 0 | 0 | 1 | 0 |
| lung | 48 | 1 | 0 | 0 | 1 | A | 1 | 95 | 1 | 1 | 0 | 0 | 0 | 0 | 0 | 0 |
| lung | 36 | 1 | 1 | 1 | 1 | B | 0 | 66 | 1 | 1 | 1 | 0 | 0 | 0 | 0 | 1 |
| lung | 36 | 1 | 1 | 1 | 1 | B | 0 | 66 | 1 | 1 | 1 | 0 | 0 | 0 | 0 | 1 |
| lung | 37 | 0 | 0 | 0 | 5 | T | 1 | 33 | 1 | 1 | 0 | 0 | 0 | 0 | 0 | 0 |
| lung | 63 | 1 | 1 | 0 | 1 | A | 0 | 87 | 2 | 1 | 1 | 0 | 0 | 1 | 0 | 1 |
| lung | 63 | 1 | 1 | 0 | 1 | A | 0 | 87 | 2 | 1 | 1 | 0 | 0 | 1 | 0 | 1 |
| lung | 43 | 0 | 0 | 0 | 40 | T | 0 | 27 | 1 | 2 | 0 | 0 | 0 | 0 | 0 | 0 |
| lung | 48 | 1 | 1 | 0 | 1 | A | 0 | 144 | 1 | 1 | 0 | 0 | 0 | 0 | 0 | 0 |
| lung | 43 | 0 | 0 | 0 | 16 | T | 0 | 71 | 1 | 2 | 0 | 0 | 0 | 0 | 0 | 0 |
| lung | 53 | 0 | 0 | 1 | 8 | H | 0 | 10 | 2 | 1 | 0 | 0 | 0 | 0 | 0 | 0 |
| lung | 48 | 0 | 0 | 1 | 50 | H | 0 | 42 | 1 | 2 | 0 | 0 | 0 | 0 | 0 | 1 |
| lung | 48 | 0 | 0 | 1 | 50 | H | 0 | 42 | 1 | 2 | 0 | 0 | 0 | 0 | 0 | 1 |
| lung | 48 | 0 | 0 | 1 | 50 | H | 0 | 42 | 1 | 2 | 0 | 0 | 0 | 0 | 0 | 1 |
| lung | 65 | 1 | 0 | 1 | 2 | B | 0 | 178 | 2 | 1 | 0 | 0 | 0 | 0 | 0 | 0 |
| lung | 56 | 0 | 0 | 1 | 1 | H | 0 | 166 | 2 | 1 | 0 | 0 | 0 | 0 | 0 | 0 |
| lung | 42 | 1 | 1 | 0 | 60 | B | 0 | 47 | 1 | 2 | 0 | 0 | 0 | 0 | 0 | 0 |
| lung | 52 | 1 | 0 | 0 | 0 | A | 0 | 46 | 2 | 1 | 0 | 0 | 0 | 0 | 0 | 0 |
| lung | 40 | 0 | 0 | 1 | 20 | H | 0 | 7 | 1 | 2 | 0 | 0 | 0 | 1 | 0 | 0 |
| lung | 46 | 0 | 0 | 0 | 0 | T | 1 | 2 | 1 | 1 | 0 | 0 | 0 | 0 | 1 | 0 |
| lung | 42 | 1 | 1 | 0 | 0 | A | 0 | 176 | 1 | 1 | 0 | 0 | 0 | 0 | 0 | 0 |
| lung | 66 | 0 | 0 | 1 | 0 | H | 0 | 64 | 2 | 1 | 0 | 0 | 0 | 1 | 0 | 0 |
| lung | 64 | 1 | 1 | 0 | 1 | A | 0 | 16 | 2 | 1 | 0 | 0 | 0 | 0 | 0 | 0 |
| lung | 68 | 0 | 0 | 0 | 15 | T | 1 | 48 | 2 | 2 | 0 | 0 | 0 | 0 | 0 | 0 |
| lung | 49 | 0 | 0 | 0 | 60 | T | 0 | 37 | 1 | 2 | 0 | 0 | 0 | 0 | 0 | 0 |
| lung | 41 | 1 | 1 | 1 | 0 | B | 0 | 163 | 1 | 1 | 0 | 0 | 0 | 0 | 0 | 0 |
| lung | 41 | 1 | 1 | 0 | 0 | A | 0 | 163 | 1 | 1 | 0 | 0 | 0 | 0 | 1 | 0 |
| lung | 44 | 0 | 0 | 0 | 10 | T | 1 | 4 | 1 | 1 | 0 | 0 | 0 | 0 | 0 | 0 |
| lung | 50 | 0 | 0 | 1 | 1 | H | 1 | 22 | 1 | 1 | 0 | 0 | 0 | 0 | 0 | 0 |
| lung | 55 | 0 | 0 | 0 | 30 | T | 0 | 86 | 2 | 2 | 0 | 0 | 1 | 1 | 1 | 1 |
| lung | 55 | 0 | 0 | 0 | 30 | T | 0 | 86 | 2 | 2 | 0 | 0 | 1 | 1 | 1 | 1 |
| lung | 38 | 1 | 0 | 1 | 0 | B | 0 | 69 | 1 | 1 | 0 | 0 | 0 | 0 | 0 | 0 |
| lung | 46 | 1 | 1 | 0 | 0 | A | 0 | 78 | 1 | 1 | 1 | 0 | 0 | 0 | 0 | 0 |
| lung | 44 | 0 | 0 | 0 | 1 | T | 1 | 4 | 1 | 1 | 0 | 0 | 0 | 0 | 0 | 0 |
| lung | 39 | 0 | 0 | 0 | 20 | T | 1 | 72 | 1 | 2 | 0 | 0 | 0 | 1 | 1 | 0 |
| lung | 62 | 0 | 0 | 0 | 1 | T | 1 | 17 | 2 | 1 | 1 | 1 | 1 | 0 | 0 | 1 |

A, luminal A, B, luminal B, H, HER-2 type, T, triple negative

| Table 2 Raw data of expression of lipid metabolism related proteins in paired primary and metastatic breast cancer | | | | | | | | | | | | |
| --- | --- | --- | --- | --- | --- | --- | --- | --- | --- | --- | --- | --- |
| met site | newHSLp | newHSLm | newPLIN1p | newPLINm | newFABP4p | newFABP4m | newCPT1Ap | newCPT1Am | newACOX1Ap | newACOX1m | newFASNp | newFASNm |
| bone | 1 | 1 | 0 | 0 | 0 | 0 | 0 | 0 | 0 | 0 | 0 | 0 |
| bone | 0 | 0 | 0 | 0 | 0 | 0 | 0 | 0 | 0 | 0 | 0 | 0 |
| bone | 0 | 0 | 0 | 0 | 0 | 0 | 1 | 0 | 0 | 0 | 0 | 0 |
| bone | 1 | 1 | 0 | 0 | 0 | 0 | 0 | 0 | 0 | 0 | 0 | 0 |
| bone | 1 | 0 | 0 | 0 | 0 | 0 | 0 | 0 | 0 | 0 | 0 | 0 |
| bone | 0 | 0 | 0 | 0 | 0 | 0 | 0 | 0 | 0 | 0 | 0 | 0 |
| bone | 1 | 1 | 0 | 0 | 0 | 0 | 0 | 0 | 0 | 0 | 1 | 0 |
| bone | 1 | 1 | 0 | 0 | 0 | 0 | 0 | 0 | 0 | 0 | 1 | 0 |
| brain | 0 | 0 | 0 | 0 | 1 | 0 | 0 | 0 | 0 | 0 | 0 | 0 |
| brain | 0 | 0 | 0 | 0 | 1 | 0 | 0 | 0 | 0 | 0 | 0 | 0 |
| brain | 1 | 0 | 0 | 0 | 0 | 0 | 1 | 1 | 1 | 0 | 1 | 1 |
| brain | 0 | 0 | 0 | 0 | 0 | 0 | 1 | 0 | 0 | 0 | 1 | 0 |
| brain | 0 | 1 | 0 | 0 | 0 | 0 | 1 | 1 | 1 | 1 | 1 | 1 |
| liver | 1 | 1 | 0 | 0 | 0 | 0 | 0 | 0 | 1 | 0 | 1 | 0 |
| liver | 0 | 0 | 0 | 0 | 1 | 0 | 0 | 0 | 0 | 0 | 0 | 0 |
| lung | 1 | 0 | 0 | 0 | 0 | 0 | 0 | 0 | 1 | 0 | 1 | 0 |
| lung | 1 | 1 | 0 | 0 | 0 | 0 | 0 | 0 | 1 | 1 | 1 | 0 |
| lung | 1 | 0 | 0 | 0 | 0 | 0 | 0 | 0 | 0 | 0 | 0 | 0 |
| lung | 0 | 0 | 0 | 0 | 0 | 0 | 0 | 0 | 0 | 0 | 0 | 0 |
| lung | 0 | 0 | 0 | 0 | 0 | 0 | 0 | 0 | 0 | 0 | 0 | 0 |
| lung | 0 | 0 | 0 | 0 | 0 | 0 | 0 | 0 | 0 | 0 | 0 | 0 |
| lung | 0 | 0 | 0 | 0 | 0 | 0 | 0 | 0 | 1 | 0 | 0 | 0 |
| lung | 0 | 0 | 0 | 0 | 1 | 0 | 0 | 0 | 0 | 0 | 0 | 0 |
| lung | 0 | 0 | 0 | 0 | 0 | 0 | 0 | 0 | 0 | 0 | 0 | 0 |
| lung | 0 | 0 | 0 | 0 | 0 | 0 | 0 | 0 | 0 | 0 | 0 | 0 |
| lung | 0 | 0 | 0 | 0 | 0 | 0 | 0 | 0 | 0 | 0 | 0 | 0 |
| lung | 0 | 0 | 0 | 0 | 0 | 0 | 0 | 0 | 0 | 0 | 1 | 0 |
| lung | 0 | 0 | 0 | 0 | 0 | 0 | 0 | 0 | 1 | 0 | 0 | 0 |
| lung | 0 | 0 | 0 | 0 | 0 | 0 | 0 | 0 | 0 | 0 | 0 | 0 |
| lung | 0 | 0 | 0 | 0 | 0 | 0 | 0 | 0 | 0 | 0 | 0 | 0 |
| lung | 0 | 0 | 0 | 0 | 0 | 0 | 0 | 0 | 0 | 0 | 0 | 0 |
| lung | 1 | 0 | 0 | 0 | 0 | 0 | 0 | 0 | 0 | 0 | 0 | 0 |
| lung | 0 | 0 | 0 | 0 | 0 | 0 | 0 | 0 | 0 | 0 | 0 | 0 |
| lung | 0 | 0 | 0 | 0 | 0 | 0 | 0 | 0 | 0 | 1 | 0 | 0 |
| lung | 0 | 0 | 0 | 0 | 1 | 0 | 0 | 0 | 1 | 0 | 1 | 0 |
| lung | 1 | 0 | 0 | 0 | 0 | 0 | 0 | 0 | 0 | 0 | 0 | 0 |

p, primary, m, metastasis
